# The Soil Geochemistry in the Beardmore Glacier Region, Antarctica: Implications for Terrestrial Ecosystem History

**DOI:** 10.1038/srep26189

**Published:** 2016-05-18

**Authors:** W. B. Lyons, K. Deuerling, K. A. Welch, S. A. Welch, G. Michalski, W. W. Walters, U. Nielsen, D. H. Wall, I. Hogg, B. J. Adams

**Affiliations:** 1 The Ohio State University, Columbus, OH 43210 USA; 2 Purdue University, West Lafayette, IN USA; 3 Hawkesbury Institute for the Environment, Western Sydney University, Penrith NSW 2751, Australia; 4 Colorado State University, Ft Collins, CO USA; 5 University of Waikato, Hamilton, New Zealand; 6 Brigham Young University, Provo, UT USA

## Abstract

Although most models suggest continental Antarctica was covered by ice during the Last Glacial Maximum (LGM) it has been speculated that endemic species of soil invertebrates could have survived the Pleistocene at high elevation habitats protruding above the ice sheets. We analyzed a series of soil samples from different elevations at three locations along the Beardmore Glacier in the Transantarctic Mountains (in order of increasing elevation): Ebony Ridge (ER), Cloudmaker (CM), and Meyer Desert (MD). Geochemical analyses show the MD soils, which were exposed during the LGM, were the least weathered compared to lower elevations, and also had the highest total dissolved solids (TDS). MD soils are dominated by nitrate salts (NO_3_/Cl ratios >10) that can be observed in SEM images. High δ^17^O and δ^18^O values of the nitrate indicate that its source is solely of atmospheric origin. It is suggested that nitrate concentrations in the soil may be utilized to determine a relative “wetting age” to better assess invertebrate habitat suitability. The highest elevation sites at MD have been exposed and accumulating salts for the longest times, and because of the salt accumulations, they were not suitable as invertebrate refugia during the LGM.

One of the most intriguing issues in Antarctic terrestrial ecology today is the biogeography of soil invertebrates and how they survived repeated glaciations during the Pleistocene Epoch^1^. The molecular genetics of various species strongly suggest that they have been continuously isolated for millions of years^2^,^3^,^4^, implying that local refugia must have existed in Antarctica, either on high elevation nunataks^5^,^6^, or in geothermally active regions^7^. Otherwise, previous ideas of Antarctic glacial history need rethinking^4^. Much work has been done in the cold, desert soils of Antarctica to understand the relationship between the geochemical properties of the soils and the distribution of organisms. From these studies, habitat suitability models have been developed and used to explain the distribution of invertebrate species in the McMurdo Dry Valleys region, and more recently, further inland in the Darwin Mountains (80°S)^8^,^9^. The harshness of these soil environments include not only cold temperatures and low water availability, but also low carbon and high salt accumulations^10^. This paper links the idea of high elevation soils from the Transantarctic Mountains (TAM) being potential refugia during glacial periods of the Pleistocene with the concept of habitat suitability.

## Results and Discussion

**Figure 1:**
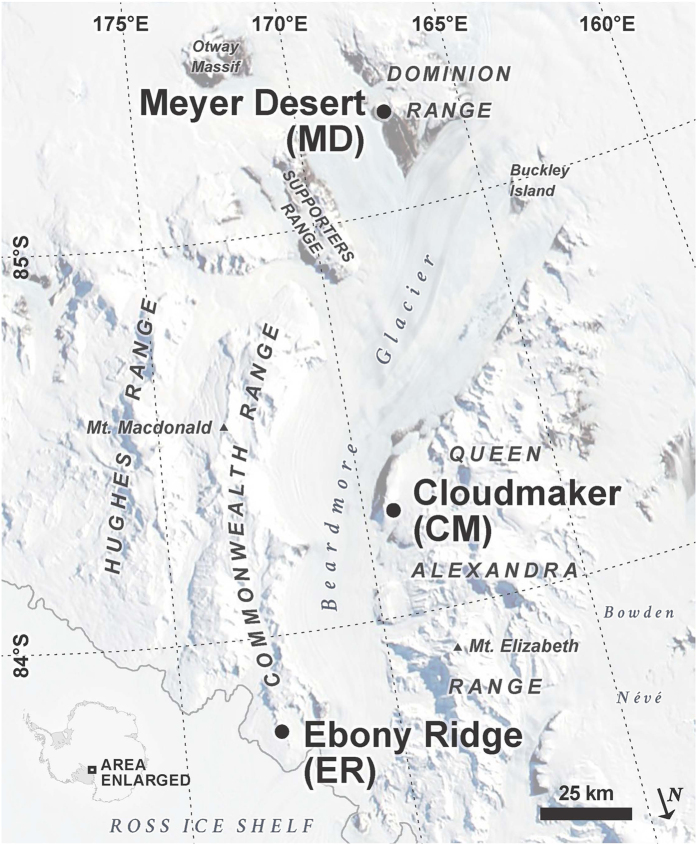
Location map of the Beardmore Glacier region of the central Transantarctic Mountains. Sampled features include Ebony Ridge (ER), Cloudmaker (CM) and Meyer Desert (MD). (Map generated using ArcMap 10.3.1 and Adobe Illustrator CS6).

Soils were collected from the drift sheets in the Beardmore Glacier region of the central Transantarctic Mountains (Fig. 1). These drift sheets have been termed, with increasing age, the Plunkett, Beardmore, Meyer and Dominion^11^. The Plunkett has a maximum ^14^C age of 3100 years, the Beardmore is Late Glacial maximum age, the Meyer is about 140,000 years, while the Dominion is thought to be pre-late Quaternary^12^. Scarrow^13^ *et al.*, recently have questioned the older ages of soils in the Beardmore region, based on exposure age measurements, and have suggested that the oldest soils may be an order of magnitude older than the ages based on soil development predictions. Recent work in the Darwin Glacier region has also indicated that the oldest soils are much older than previously thought^14^.

In this work, samples were collected from three locations: Ebony Ridge (ER) in the Mt. Kyffin region, the Cloudmaker (CM), and the Meyer Desert (MD) (Fig. 1; Table 1). The organic and inorganic carbon and total N and P concentrations, as well as the percentage of sand and silt of these soils are shown in Table 1. The total C and N concentrations are within the same range as previously reported^13^,^15^, and are very low; <0.15% for N and <0.5% for C.

**Figure 2:**
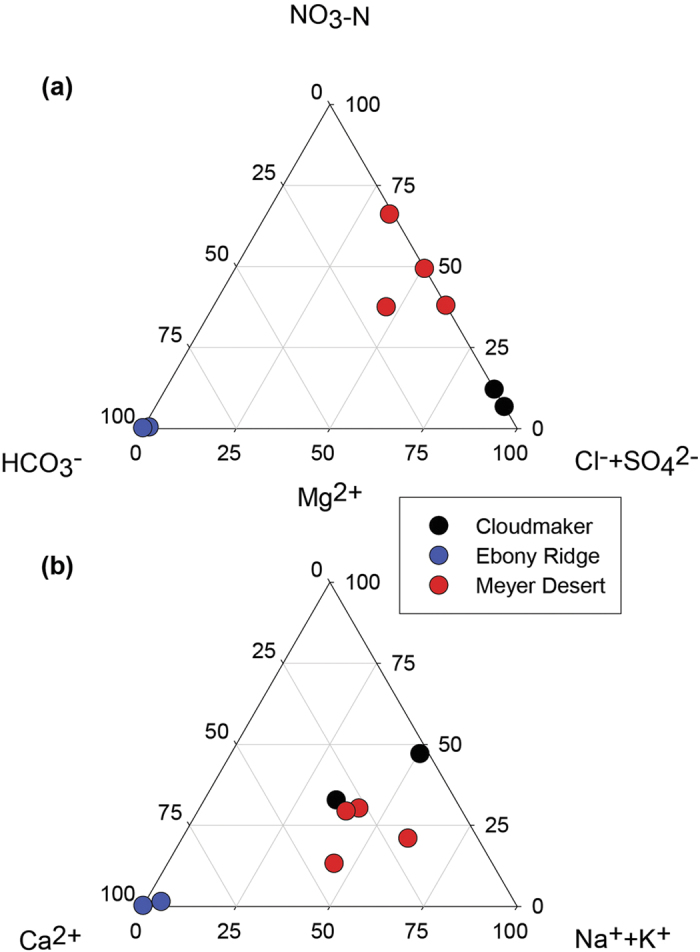
Ternary plots of (**a**) anion and (**b**) cations concentration (milliequivalents/liter) from the water leaches of TAM soils from Cloudmaker (CM), Ebony Ridge-Mt. Kyffin (ER) and the Meyer Desert (MD).

**Figure 3:**
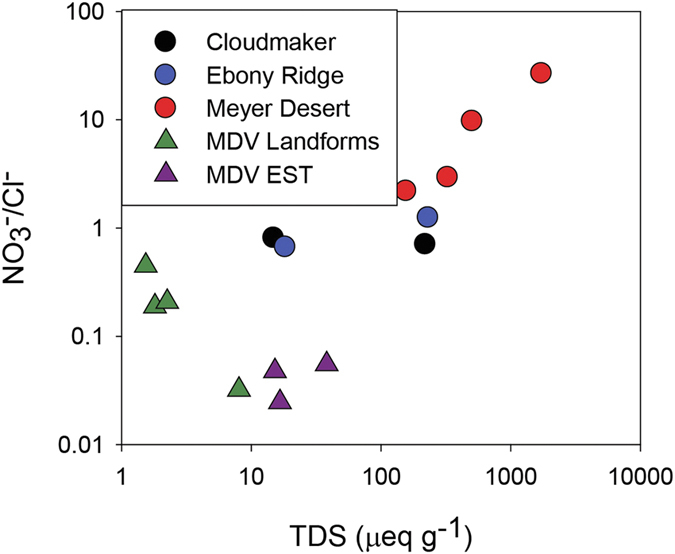
NO_3_/Cl versus TDS for 1:5 soil:water leaches of TAM soils. Data from the McMurdo Dry Valleys (MDV) sites^37^ are plotted for comparison.

**Figure 4:**
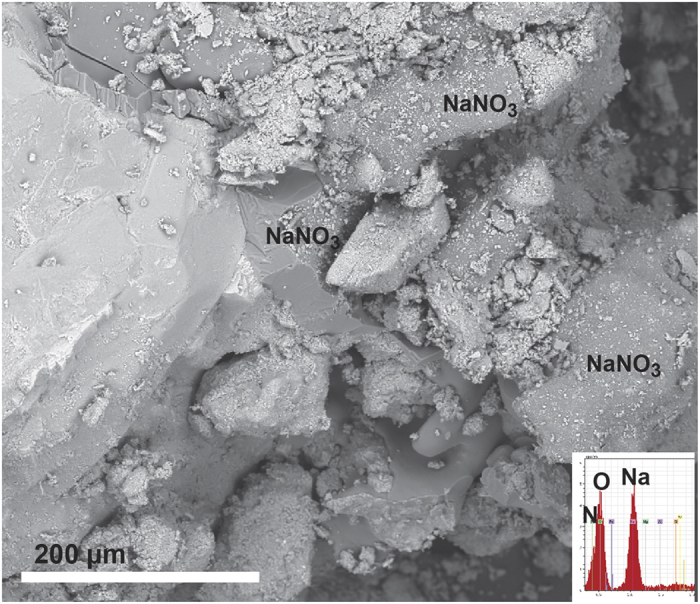
SEM image of Meyer Desert soil sample (MDBJA4). NaNO_3_ is evident as a dark phase in the BSE image. Insert shows EDS spectra of a typical spot analysis of NaNO_3._

The water:soil leachate data for both the cations and the anions are shown as ternary plots in Fig. 2. The two soils from CM and the four from MD show a range of cation distributions but the ER samples are dominated by Ca^2+^. The anion plot shows a clear separation between the three sites with ER dominated by HCO_3_^−^, CM by Cl^−^ + SO_4_^2−^ and MD by NO_3_^−^ or NO_3_^−^ and Cl^−^ + SO_4_^2−^. The predominance of NO_3_^−^ in the soluble salts of the MD soils can be seen in Fig. 3, as the highest TDS samples also have the highest NO_3_^−^/Cl^−^ ratios. The results are similar to what has been observed in other soil studies, both in the central TAM and other parts of Antarctica, in that the highest elevation soils have very high nitrate concentrations, and dominate the soluble anion concentrations^11^,^15^,^16^. The nature of the NO_3_^−^ in these soils can be demonstrated in back scattered electron (BSE) SEM images, as NaNO_3_ is clearly observable as a dark phase in the MD soils (Fig. 4). Composition of this phase was confirmed by energy dispersive spectroscopy (EDS) spot analysis. The stable isotopic signature of the soluble NO_3_^−^ is similar to values observed from soils from the McMurdo Dry Valleys region (Table 2). The elevated δ^18^O and Δ^17^O values indicate that the nitrate is derived 50% from tropospheric transport of photochemically produced HNO_3_, and ~50% from HNO_3_ formed in the stratosphere^17^. The highly enriched oxygen values are attributed to oxidation reactions in the polar atmosphere involving ozone, and indicate that the nitrate was not produced by biological processes. It is not surprising that the nitrate is not derived from biological processes due to the very low organic carbon in the soil.

Previous work in the TAM has indicated that altitude, not latitude, may be far more important in soil development^13^,^16^. The amount and frequency of moisture addition to these soils have significant effects on the total salt accumulation as well as the elemental distribution of the salts. The high concentrations of nitrate in the MD samples imply there has been little to no moisture input into these soils for a significant amount of the time they have been exposed. Because salts such as NaNO_3_ are extremely soluble, we can calculate the potential age of the last significant moisture flush, “wetting age”, through the NO_3_-rich soil using the following approach. We can take the total water-leachable NO_3_^−^ in the top 10 cm of this soil and divide this by the NO_3_^−^ flux in the snow from the nearby Dominion Range ice core^17^. A wetting age of between 750,000–850,000 years BP is computed for the Meyer Desert.

If we use the same NO_3_^−^ flux for the Dominion range determined by Lyons *et al.*^17^ for the CM and ER sites, the accumulated NO_3_^−^ yields wetting ages between 20–23,000 years at CM and between 56 and 63 years for ER. These findings suggest that the lower ice-free locations along the Beardmore receive more precipitation and/or melt. These calculations include a number of important assumptions, including that the NO_3_^−^ input into the snow has remained constant over this time interval, and that there has been no post-depositional loss of NO_3_^−^ either from the snow (which would impact the original nitrate flux calculation of Lyons *et al.*^17^) or from the soil. Data suggest that there can be NO_x_ loss from the Antarctic snow pack^18^, and others have argued for the possibility that NO_3_^−^ can be added to TAM soils through a downslope transfer from the plateau^15^. It has been demonstrated that the lost NO_3_^−^ from Antarctic snow is the lighter isotope^19^, which is what is accumulating in the soils, so we cannot rule out that a portion of the NO_3_^−^ in these soils is from recycling of NO_3_^−^ from nearby snow. In any effect, this “wetting age” is a best estimate based on the available information. It is interesting to point out that the Plunkett and Beardmore drifts were estimated to have a maximum age of 23,800 years^11^, but as noted above, recent cosmogenic exposure ages^13^ from moraines in these drifts yield much older ages (300,000–500,000 years).

**Figure 5:**
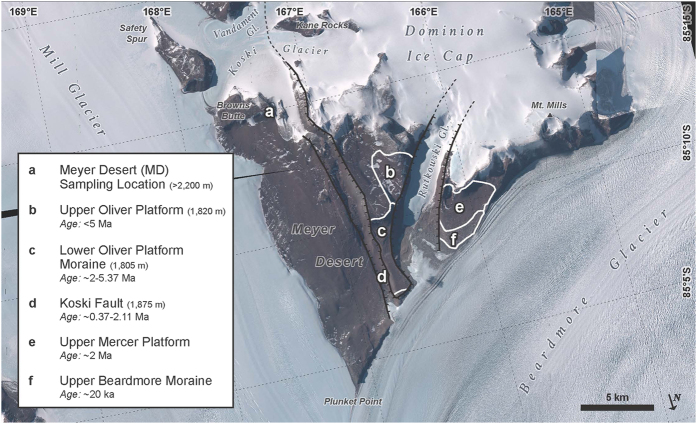
Map of the Meyer Desert at the confluence of the Mill and Beardmore glaciers. Box inset describes the locations, elevations and/or relative age of the formations in the area (modified from Ackert & Kurz^20^). The samples described in the present study are from Browns Butte (**a**). (Map generated using ArcMap 10.3.1 and Adobe Illustrator CS6).

The “wetting age” of our sample locations and their elevation during the LGM is important for determining their potential to serve as refugia. Our MD samples came from the area adjacent to Brown’s Butte near the upper bench of the Oliver Platform by the Koski Fault at the elevation of above 2200 m (our highest elevation sample is at 2551 m; Figs 1 and 5, Table 1). Ackert and Kurz^20^ reported a number of cosmogenic exposure age dates from this area but none above 1900 m. Their ages range from ~2 to 5.37 Myr at elevations of 1805–1820 m, while the Koski Fault age data range from 0.37 to 2.11 Myr (at elevations of 1875–1950 m). So our highest elevation samples probably represent Sirius Group sediments that sit on an erosional surface above the local bedrock and are significantly older than lower elevation soils^21^. The soil at 2551 m could have been a candidate for a potential refugium location not only during the LGM, but for all of the glaciations during the Pleistocene, as a moraine between the lower Oliver and upper Oliver Platform dates at ~5 Myr^20^ (Fig. 5).

Recent evidence indicates that both the West and the East Antarctic Ice Sheets (WAIS and EAIS, respectively) were at lower elevations in their interiors during the LGM than previously thought^22^,^23^. Denton and Hughes^24^ have argued that the surface elevations of the EAIS inland of the TAM were lower than present during the LGM except near the outlet glaciers, which were dammed by grounded ice in the Ross Sea. This thickening of the outlet glaciers led to slightly increased surface elevations at the heads of the glaciers in the TAM but substantial rises in ice surfaces at the mouths of the glaciers near Ross Ice Shelf 24. However, exposure age dating in the Darwin Glacier area indicates that even the increases near the ice shelf were probably lower than previous estimates^14^. Denton and Hughes^24^ suggest a maximum inland ice-rise of the Beardmore Glacier of 35–40 m, but increases of as much as 1300 m in the Mt. Kyffin (Ebony Ridge) region. Taking all the evidence into account it has become clear that even during glacial maxima times, high-elevation ice-free areas existed even at these very high latitudes. The high elevation areas in the TAM could have served as refugia during the times when the lower elevation regions were covered with ice or in southern Victoria Land, when large lakes covered the valley floors^25^.

**Figure 6:**
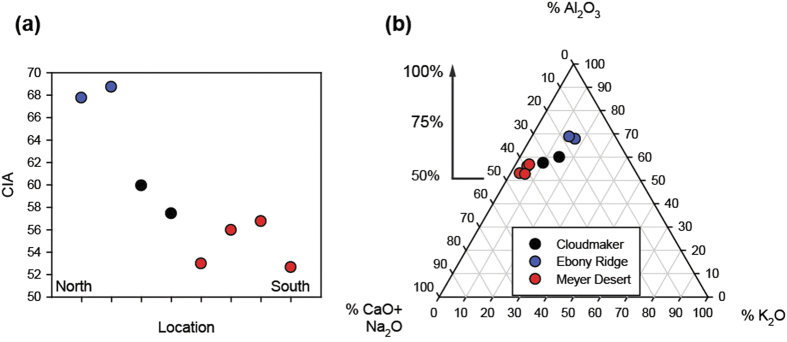
(**a**) Chemical Index of Alteration (CIA) as a function of relative distance (north to south) and (**b**) ternary diagram of major oxides for TAM soils.

We have computed the Chemical Index of Alteration, CIA, using the major oxide data for each soil sample. This parameter has been utilized to determine the extent of chemical weathering in sedimentary rocks, sediments and soils^26^, and is determined by this equation:


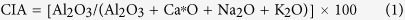


where Ca*O is the non-carbonate calcium present in the sample. As aluminosilicate minerals become more intensely weathered, the alkaline earths and alkali metals are lost and the CIA approaches 100%. Fresh granites/granodiorites have CIA values between 45–55^26^, and mean continental crust is 58^27^. The CIA values from these soils are plotted vs relative latitude in Fig. 6. Clearly the MD data show much lower values, more representative of unweathered material, than the ER samples, which appear highly weathered. The CM samples lie in between.

The previous compilation of data indicates that nitrogen concentrations increase with elevation, and with age in most Antarctic soils. Inorganic carbon (IC) increases with precipitation, and in general, organic carbon concentrations do not change with age of the soils^15^. The highest IC we observed is at ER, where we also see the highest water-leachable HCO_3_^−^ and Ca^2+^ in the soil (Table 1). IC was only observed in one of the four MD samples. The low solid OC/P ratios in these soils are due in very large part to the low OC values. The high water-leachable N:P ratios in the CM and MD sites are due to the very high inputs of atmospherically derived nitrate as described above, and as indicated by the isotopic data of the nitrate. These data indicate that with the exception of ER, these soil ecosystems are very P limited. This is also the situation for pre-LGM tills in southern Victoria Land^28^.

Hence both the leachate data, with its low concentrations of soluble salt and predominance of Ca^2+^ and HCO_3_^−^, and the bulk soil analysis, with a much higher CIA, suggest that ER has been more impacted by liquid water than the other sites, and that water has leached the soluble salts and caused extensive chemical weathering. In contrast, the MD samples have high concentrations of the most soluble salts, such as NaNO_3_, and little chemical weathering has taken place. Our ER samples are very close to, and at the same elevations, as a hotspot of lichen diversity described by Green *et al.*^29^. This location yielded 28 different lichen species and their occurrence has been attributed to “relicts” that have survived on-site through the glacier maxima periods. These authors suggested that these relict populations at the ER site could be as young as 200 Kyr, but they could be much older as well. Nonetheless, this site is clearly a more suitable habitat for life due to the abundance and/or frequency of liquid water as evidenced by the geochemical information.

### When is a Refugia Not a Refugia?

As noted in the introduction of this paper, there are significant observational and molecular/genetic data to suggest that much of the invertebrate fauna found in the continental soils of Antarctica today have survived through the glacial maxima periods of the Pleistocene^4^,^6^. In order for these species to persist over this time period, there would be a need for habitat continuity for each species^6^, and these habitats would probably have to be at higher elevations that the WAIS and the EAIS were unable to overtop as they advanced oceanward. However, different soil factors and climate have been found to define suitable habitats for particular species, and hence community composition in Antarctic soils^8^,^10^. Younger soils with less salt have a more complex community structure^9^. High salt concentrations, especially nitrate, can strongly inhibit the survival of the endemic nematodes including, *Scottnema lindsayae* and *Plectus antarcticus*^30^. *Scottnema lindsayae* previously collected from the Beardmore region show no discernable morphological or ITS1 rDNA sequence variation from those of the McMurdo Dry Valleys, suggesting relatively frequent and widespread dispersal within and among suitable habitats^31^. Clearly the MD sites with their very high nitrate concentrations would not be good refugia candidates, even though they were ice-free throughout the Pleistocene.

So the question becomes, where were high altitude refugia that also had suitable habitats? It is now very clear that abiotic spatial gradients that cut across landscape units (e.g. streams to soils) and elevational gradients, rather than latitudinal gradients, greatly impact the distribution of soil invertebrates and other organisms in Antarctic soils^8^,^9^,^13^,^29^,^32^,^33^. These gradients in abiotic properties can exist over very short distances^13^. This local patchiness may be related to the slow lowering of the ice sheet via sublimation and/or differential snow accumulation and consequent melting through solar radiation absorption by the surrounding low albedo soil. Scarrow *et al.*^13^ point out that this small-scale variation in the abiotic properties of these high elevation soils cannot be effectively mapped. Thus the overlap of ice-free refugia and suitable habitat may have only existed on such scales that make it extremely difficult to map and hence identify. If this is indeed the case, the exercise of predicting the past locations of refugia will require describing both past soil conditions and glacier/ice elevation on a much more detailed scale.

## Conclusions

Soil samples analyzed from the Beardmore Glacier area of the central Transantarctic Mountains (TAM) of Antarctica have very different geochemical characteristics. These differences are driven by the present or recent past availability of liquid water that both leaches soluble salts and acts as a chemical weathering agent of the soils. The soils with the highest concentrations of salt do not provide suitable habitat for metazoan life even though they were undoubtedly ice-free during the Pleistocene glaciations. These data and previous soil biogeochemical/ecological research at lower latitudes in Victoria Land suggest that gradients in abiotic properties exist on many scales in Antarctic soils. Some of these variations, including those most closely associated with habitat suitability, occur at very small spatial scales, complicating the interpretation of past biogeochemical patterns and the identification of LGM refugia.

## Methods

Multiple soil samples were collected from the top 10 cm at each location using clean plastic implements and placed into sterile plastic bags (Whirlpak®, Fort Atkinson, WI, USA) and transported in insulated coolers to the Crary Laboratory, McMurdo Station. In the laboratory, samples were gently homogenized and subsampled for geochemical analysis under a laminar flow hood.

Aliquots of dried soil were leached with deionized water (DI) using a 1:5 soil:water ratio^13^. The leachates were filtered with a 0.4 μm polycarbonate membrane filter and analyzed for major cations and anions (Na^+^, K^+^, Ca^2+^, Mg^2+^, Cl^−^, SO_4_^2−^, NO_3_^−^) using ion chromatography. Phosphorous, as phosphate, was analyzed using a Skalar Nutrient Analyzer. The ion chromatographic data had a precision of **≤** 5%. Bicarbonate was determined by subtracting the sum of Cl^−^, NO_3_^−^ and SO_4_^2−^ equivalents from the sum of Na^+^, K^+^, Ca^2+^, and Mg^2+^in equivalents^34^. Another subset of bulk soil was analyzed for major element oxides, including P_2_O_5,_ using X-ray fluorescence according to the lithium borate dissolution technique^35^. USGS standard reference materials were measured similarly and were within 12% of the reported values. Total carbon, organic carbon and total nitrogen were measured on bulk samples using a Leco C/N analyzer. The percentage of sand and silt grain size of subsets of soils was also determined using standard sieving techniques. All of these analyses were done at The Ohio State University. Several samples were analyzed using an FEI Quanta FEG field emission SEM. Samples were affixed to carbon tape on a stub and coated with Au-Pd before analysis. Finally, another subset of samples was leached with water and the nitrate in the leachate was analyzed for δ^15^N, δ^18^O, and δ^17^O at Purdue University using the techniques of Michalski *et al.*^36^.

Maps (Figs 1 and 5) were generated using ArcMap 10.3.1 and Adobe Illustrator CS6.

## Additional Information

**How to cite this article**: Lyons, W. B. *et al.* The Soil Geochemistry in the Beardmore Glacier Region, Antarctica: Implications for Terrestrial Ecosystem History. *Sci. Rep.* **6**, 26189; doi: 10.1038/srep26189 (2016).

## Figures and Tables

**Table 1:** Samples collected from the TAM sites, size fraction, percent organic carbon, percent inorganic carbon, organic carbon/phosphorus, nitrogen /phosphorus.

	Elevation	% sand	% silt	% OC	% IC	OC/P	N/P
	(m)					(solid)	(leach)
**Ebony Ridge - Mt. Kyffin (ER)**							
KYUN2	488	21	14	0.09	0.18	3	100
KYUN7	547	41	37	0.33	0.13	11	17
**Cloudmaker (CM)**							
CMUN1	1635	72	1	0.09	0.04	6	163
CMUN8	1973	51	4	0.10	0.04	5	1.8 × 10^4^
**Meyer Desert (MD)**							
MDBJA4	2310	91	5	<0.08	<0.08	<7	1.8 × 10^5^
MDBJA3	2343	80	5	<0.08	<0.08	<8	4.2 × 10^4^
MDBJA2	2370	80	1	<0.08	<0.08	<8	2.3 × 10^3^
MDBJA1	2551	54	2	<0.08	<0.08	<7	2.5 × 10^5^

**Table 2:** Stable isotopes of oxygen and nitrogen in nitrate at the Meyer Desert.

	δ^18^O ‰	δ^17^O ‰	Δ^17^O	δ^15^N ‰	elevation (m)
MDBJA4	72.0	71.1	33.5	2.8	2310
MDBJA3	66.6	67.7	32.9	5.3	2343
MDBJA2	59.4	59.3	28.4	8.8	2370
MDBJA1	71.2	69.4	32.3	1.8	2551
